# Virulence among different types of hypervirulent *Klebsiella pneumoniae* with multi-locus sequence type (MLST)-11, Serotype K1 or K2 strains

**DOI:** 10.1186/s13099-021-00439-z

**Published:** 2021-06-22

**Authors:** Tsui-Chin Wang, Jung-Chung Lin, Jen-Chang Chang, Ya-Wen Hiaso, Ching-Hsun Wang, Sheng‑Kung Chiu, Chang-Phone Fung, Feng-Yee Chang, L. Kristopher Siu

**Affiliations:** 1 National Institute of Infectious Disease and Vaccinology, National Health Research Institute, Zhunan, Miaoli Taiwan; 2 Division of Infectious Diseases and Tropical Medicine, Department of Internal Medicine, Tri-Service General Hospital, National Defense Medical Center, Taipei, Taiwan; 3 Cathay General Hospital, Shijhih, New Taipei City, Taiwan; 4 Graduate Institute of Basic Medical Science, China Medical University, Taichung, Taiwan

**Keywords:** *Klebsiella pneumonia*, Hypervirulence, Serotype, MLST

## Abstract

**Background:**

Two different types of hypervirulent *K. pneumoniae* (HvKp), the MLST-11 and serotype K1/K2 strains, have been frequently described in recent studies. Although these two types of strains were described to be HvKp, their virulence was not compared. In this study, in vitro and in vivo approaches were used to assess differences in virulence.

**Materials and methods:**

A total of twenty-nine isolates, including 6 strains of each of serotype K1 and K2 isolates and 17 strains of ST11 isolates, were selected for this study. Phenotypic tests of virulence were performed by the string test and analysis of the virulent associated genes was detected by PCR. In vitro models of serum resistance and phagocytosis were used as the parameters to assess the virulence. In-frame deletion of virulence-associated genes was performed to study their contributions to virulence. The median lethal dose, i.e., the LD_50_, in mice was determined following IP injection.

**Results:**

Although serotype K1 and K2 strains and ST11 isolates had similar virulence gene profiles, the ST11 isolates showed less serum and phagocytic resistance than the serotype K1/K2 isolates. The mouse lethality test revealed that all ST11 isolates were unable to cause lethality, even at > 10^7^ CFU, while serotypes K1 and K2 showed an LD_50_ at ≤ 10^3^ CFU. Aerobactin or capsule knockout mutants exhibited a lower LD_50_ than the parental strain, while capsule mutants showed a more significant decrease in LD_50_.

**Conclusion:**

Since there was a significant difference in virulence levels between the two types of HvKp when assessed in in vitro and in vivo models, it may be better to use the designation "HvKp" for some strains based on animal studies to avoid confusion. Virulence and non-virulence could be analysed in a relative manner, especially in comparison studies.

**Supplementary Information:**

The online version contains supplementary material available at 10.1186/s13099-021-00439-z.

## Background

*Klebsiella pneumoniae* is a gram-negative bacterium and an opportunistic pathogen that can cause both community-acquired and nosocomial infections. Recently, *K. pneumoniae* has been categorized into classic *K. pneumoniae* (cKp) and hypervirulent *K. pneumoniae* (HvKp) according to differences in virulence. HvKp is differentiated from cKp by its ability to develop subsequent metastatic spread or to present in multiple sites of infection [1]. Metastasis, such as liver abscess in patients with meningitis or endophthalmitis [2–4], has been well described previously. Generally, cKp is often found and described in nosocomial isolates, while HvKp was initially described in patients with community-acquired liver abscesses from Taiwan, Singapore, Hong Kong [5] and South Korea [6]. Subsequently, the term HvKp was recently used for nosocomial isolates with a common multi-locus sequencing type (MLST) 11 [2, 7]. This ST11 isolate was first identified to contribute to a significant outbreak of *K. pneumoniae* with carbapenem resistance in a local hospital [7]. Alarmingly, this HvKp strain is multidrug resistant (MDR) and exhibits resistance to drugs such as carbapenems. This MDR-HvKp is difficult to treat [3], and pneumonia patients infected with this type of strain may die due to multiorgan failure or septic shock [2, 7]. MDR-HvKp encodes virulence genes, including several siderophore genes (*entB*, *iroN*, *iucA*, and *iutA*) and one capsular polysaccharide regulator gene (*rmpA*) that contribute to capsule expression. In addition, this MDR-HvKp carries the *bla*_*kpc2*_ gene, resulting in a carbapenem-resistance pathotype, which is consistent with other classes of drug resistant strains. In addition to MDR-HvKp, another type of HvKp has also been frequently mentioned in the literature [8]. The clinical characteristics of infection with this type of HvKp are different. This type of HvKp is mostly derived from community-acquired infections, especially liver abscesses and pneumonia. Diabetes is the only human risk factor for liver abscess patients with this type of HvKp infection. Although some virulence factors from liver abscess isolates are the same as those from MDR-HvKp, the serotype prevalence is unique. Over 70–80% of isolates from liver abscesses are from serotypes K1 and K2. To the best of our knowledge, complications of endophthalmitis or meningitis all result from serotype K1 or K2 isolates [9–11], while HvKp with ST11 is usually serotype K20, K47 or K64 [7, 12, 13]. Notably, these two types of *K. pneumoniae* are both referred to as HvKp in the literature (Table 1). However, there is no study comparing the virulence of these two types of HvKp. Thus, it may be difficult to define the term hypervirulence. In the present study, we compared these two types of strains named HvKp and investigated whether there is any difference in virulence that could be used to redefine hypervirulence.

**Table 1:** Previous publications that used the term "hypervirulent *Klebsiella pneumoniae*" in the past 3 years identified by a search of Medline

Year	Hypervirulent *K. pneumoniae*		
	None^a^	Serotype K1 or K2^a^	ST11^c^
2019	62	19	6
2018	70	22	10
2017	36	18	7

^a^Number of publications found in Medline with the term "hypervirulent *K. pneumoniae*" only

^b^Number of publications found in Medline with the term "hypervirulent *K. pneumoniae*" with serotype K1 or K2

^c^Number of publications found in Medline with the term "hypervirulent *K. pneumoniae*" with ST11

## Methods and materials

### Serotyping and MLST for *K. pneumoniae* isolates

Isolates were collected from a previous study [14]. Serotyping of K1 and K2 isolates was performed by a rapid testing cassette and PCR [15, 16]. For non-serotype K1/K2 from rapid testing, serotyping was performed by using *wzi* and *wzc* sequencing [17, 18] and capsule-specific primers for serotyping [19]. MLST with seven housekeeping genes (*gapA, infB, mdh, pgi, rpoB, phoE* and *tonB*) was performed via PCR using corresponding primer pairs [20]. A total of 9 isolates were selected for this study.

### Virulence gene profiling and the string test

Six genes, i.e., *entB, iroN, iucA, iutA, clbA,* and *rmpA,* were selected for detection in the *K. pneumoniae* isolates. The primers used for these virulence genes are listed in Additional file 1: Table S2 [2, 21, 22]. Bacterial DNA was prepared by suspending one loopful of fresh colonies in 50 µl of DNAzol (DNAzol® DIRECT, Molecular Research Center, Inc. Cincinnati, OH) and heating the mixture at 95 °C for 10 min. AmaR OnePCR (amaR OnePCR, GeneDireX®, Vegas, NV) was used as the PCR mixture, and the amplification procedure was performed according to the manufacturer’s protocol.

The string test was performed as a phenotypic method to assess virulence [4]. Isolates were streaked on a blood agar plate and cultured overnight. Hypermucoviscosity criteria were defined as previously described [7, 11, 23]. The colony was stretched with an inoculation loop to measure the visible string, and a string longer than 5 mm was considered to indicate a hypermucoviscosity phenotype.

### Generation of aerobactin and capsule knockout mutants by in-frame deletion

In-frame deletion mutagenesis was used to generate mutants for virulence studies [24]. In brief, the primer sets iucA-AR and iucA-BF were used for *iucA* deletion construction (Additional file 1: Table S1), and these primer sets were complementary to each other. The resulting PCR fragment was digested with XbaI and SacI and then cloned into the puTkmy-MCS plasmid for the 799 and 794 strains and the puTkmy-MCS-zeocin plasmid for the 3016 strain. The single-crossover strains were selected from green inositol-nitrate-deoxycholate (BIND) plates. The transconjugant was selected and verified by PCR with primer sets [25] (Additional file 1: Table S2).

### In vitro virulence assessment by neutrophil phagocytosis and the serum resistance assay

A neutrophil phagocytosis assay was performed as previously described [26]. The procedure for the isolation of neutrophils and pooled serum from healthy volunteers was approved by the Research Ethics Committee of the National Health Research Institute (number: EC1061212-E).

The serum bactericidal assay was performed as described in Podschun’s study and our previous study with modifications [24, 27]. In brief, the bacteria were streaked on Mueller–Hinton agar (MHA) plates and incubated overnight to collect a single colony for inoculation in brain heart infusion (BHI) broth until the optical density at 600 nm (OD_600_) measured 0.4. After dilution with phosphate-buffered solution (PBS) from 10^8^ to 10^6^, the bacteria were combined with 750 µl of human serum and incubated for 0, 1, 2, or 3 h. The mixtures incubated for each time period were serially diluted, and 10^–3^, 10^–4^, and 10^–5^ dilutions were streaked on the MHA plate for visual bacterial counts. The results are expressed as the percentage of inoculum, and responses in terms of viable counts were graded from 1 to 6 as described previously [24, 27]. Each strain was tested at least three times. A strain was considered serum-resistant or serum-sensitive if the grading was the same in all experiments. Each isolate was classified as highly sensitive (grade 1 or 2), intermediately sensitive (grade 3 or 4), or resistant (grade 5 or 6).

### Mouse lethality test

Male BALB/c mice aged 6–8 weeks (n = 6/strain) were used for lethality testing. They were purchased from the National Laboratory Animal Center, Taiwan, and housed in the National Defense Medical Center Laboratory Animal Center. The animal protocols performed in this study were reviewed and approved by the Institutional Animal Care and Use Committee of the National Defense Medical Center (IACUC-19–271) and National Health Research Institute (NHRI) (NHRI-IACUC-107009-A).

Strains were subjected to an acute lethality test in a mouse model. The day before the experiment, the bacteria were streaked on MHA plates and grown in an incubator overnight. The bacteria were transferred to BHI broth and kept in an incubator until an OD_600_ of 0.9 was achieved. After centrifugation and serial dilutions with PBS, the bacteria were randomly intraperitoneally (IP) injected into the mice (0.1 ml/mouse, acute injection). Within 14 days of observation, the lethality and virulence of the bacteria were assessed by determining the mouse survival rate. Food and water were provided ad libitum*,* and the cages and bedding were changed once a week. This experiment was performed in duplicate to confirm the virulence degree of the bacteria. The LD_50_ was calculated using SigmaPlot version 7.0 from SPSS Inc. (Chicago, IL).

### Statistical analysis

Statistical significance was determined by two-tailed t test with GraphPad Prism for the phagocytosis study. P ≤ 0.05 was considered statistically significant.

## Results

### MLST, serotyping and virulence-associated gene analysis of the isolates selected for this study

MLST of 29 isolates revealed that a total of 17 isolates were ST11 and 6 isolates were ST23. The remaining 3, 2 and 1 isolates were ST65, ST86 and ST373, respectively (Table 2). Serotyping by rapid test cassette and PCR showed that 6 of each isolate belonged to serotype K1 or K2. Furthermore, *wzi* and *wzc* sequencing by PCR showed that non-K1 and K2 isolates had *wzi* and *wzc* sequences similar to those of serotypes K20, K47 and K64. Subsequent PCR confirmation with serotype-specific primers for K20, K47 and K64 showed that all isolates belonged to serotypes K20, K47 and K64 (Table 2). The expression of the virulence-associated genes *clb, entB*, *iroN*, *iucA*, *iutA*, and *rmpA* was assessed in all isolates from serotypes K1, K2 and ST11 (K20, K47 and K64). Except for 4 K2 isolates, which lacked *clb*, all serotype K1 and K2 isolates contained 6 virulence-associated genes, i.e., *clb, entB*, *iroN*, *iucA*, *iutA* and *rmpA*. The serotype K2 isolates that lacked *clb* included one each of those with ST65 and ST373 and 2 with ST86. Except for one isolate with K64 that carried *iucA*, ST11 isolates with serotypes K47 and K64 contained only one virulence-associated gene, namely, *entB*. In contrast, ST11 with serotype K20 isolates contained 5 virulence-associated genes, i.e., *entB*, *iroN*, *iucA*, *iutA*, and *rmpA* (Table 2)*.* Thus, a total of 5 different serotypes of isolates with 7 different MLSTs were included in further assessment of virulence in in vitro and in vivo models (Table 2).

**Table 2:** Serotype, MLST and virulence-associated gene detection of the isolates in this study

No. of strains	Serotype	MLST	Virulence gene profiles						Serum resistance: (R, I, S)^a^	LD_50_ (CFU range)
			*clb*	*entB*	*iroN*	*iucA*	*iutA*	*rmpA*		
6	K1	23	+	+	+	+	+	+	R	> 2 × 10^2^–< 2 × 10^3^
2	K2	65	+	+	+	+	+	+	R	< 10^2^–2.4 × 10^2^
1	K2	65	−	+	+	+	+	+	R	< 4 × 10^2^
2	K2	86	−	+	+	+	+	+	R	< 7.3 × 10^2^
1	K2	373	−	+	+	+	+	+	R	< 1 × 10^2^
6	K20	11	−	+	+	+	+	+	S	> 10^7^
5	K47	11	−	+	−	−	−	−	S	> 10^7^
5	K64	11	−	+	−	−	−	−	S	> 10^7^
5	K64	11	−	+	−	−	+	−	S	> 10^7^

^a^*R* resistance, *I* intermediate resistance, *S* susceptible. Serum resistance was graded according to the method described in the materials and methods

### String test for serotype K1, K2 and ST11 isolates and their derived mutants

**Fig. 1:**
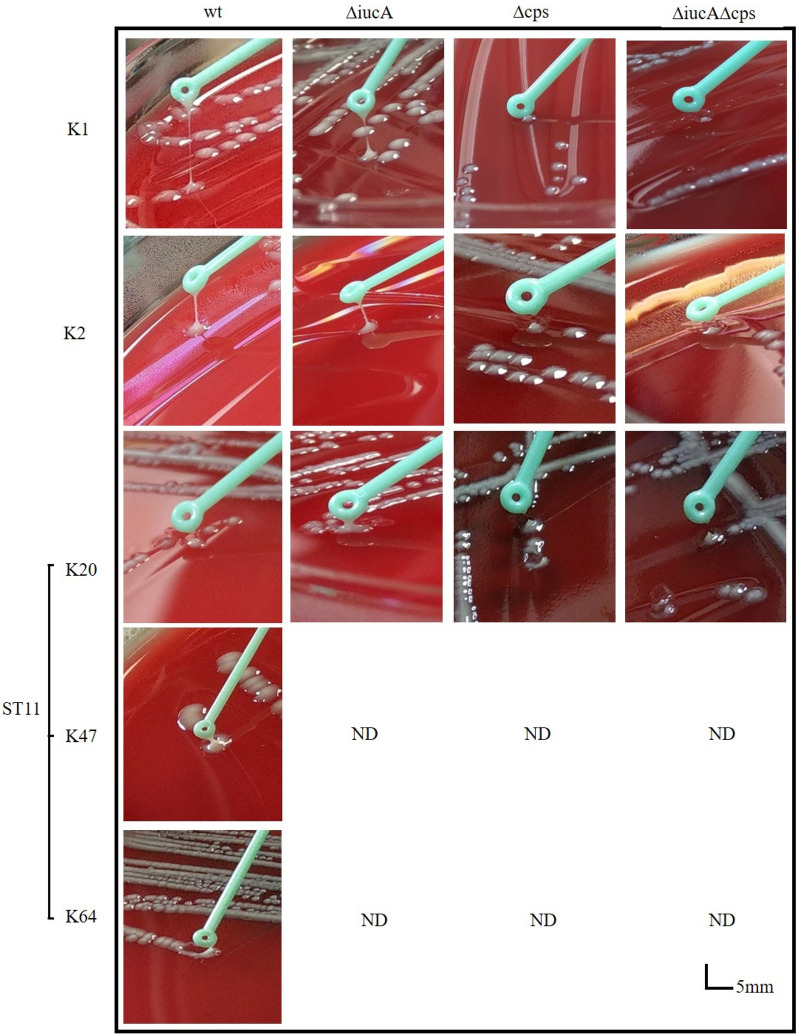
String test results for HvKp isolates with serotype K1/K2 and ST11 with serotype K20, K47 and K64. The mutants included Δ*iucA,* Δ*cps* and Δ*iucA/*Δ*cps* double mutants derived from a serotype K1/K2 isolate and a ST11 isolate with serotype K20

Since iron acquisition-related systems and capsule production-related systems have been described as the major factors contributing to hypervirulence and because the string test was suggested as a means of rapidly determining the virulence degree of unknown isolates [11, 23], we performed the string test as an initial assessment of virulence. Our results indicated that parental serotype K1/K2 isolates and their iron acquisition-related *iucA* mutants were positive (had a string longer than 5 mm) in the initial screening by the string test. The string test indicated that *iucA* mutants did not contribute to hypermucoviscosity, while *cps* mutants showed loss of mucoviscosity. Isolates of ST11, including parents and mutants, were negative in the string test (Fig. 1).

### Serum resistance, neutrophil phagocytosis and mouse lethality between serotype K1/K2 and ST11 isolates

**Fig. 2:**
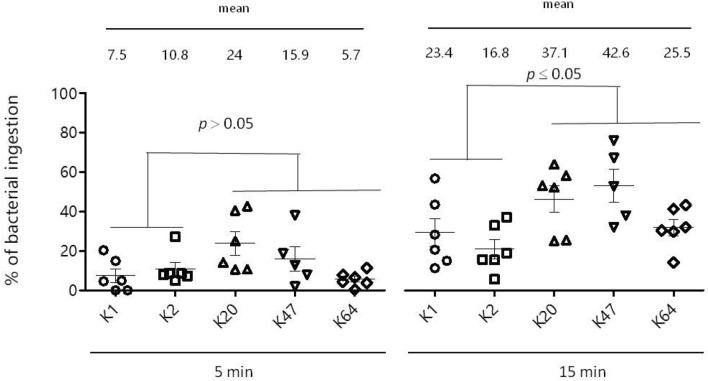
Neutrophil phagocytosis among HvKp from wild-type isolates of serotype K1/K2 and ST11 with serotype K20, K47 and K64 isolates

All serotype K1 and K2 isolates were resistant to serum complement killing, while all ST11 isolates were serum-susceptible. In comparing mouse lethality, serotype K1/K2 isolates had a low LD_50_ ranging from < 10^2^ to 2 × 10^3^ CFU, while all ST11 isolates with serotypes K20, K47 and K64 showed a high LD_50_ with > 10^7^, indicating avirulence of all ST11 isolates (Table 2). Neutrophil phagocytosis with human serum opsonization was used to assess the bacterial response to the first-line human defence mechanism. Among all isolates, serotype K1 or K2 isolates, which had an adjusted zero at 0 min, were generally more phagocytosis-resistant than ST11 isolates with serotypes K20, K47 and K64 (Fig. 2).

### Neutrophil phagocytosis and mouse lethality among parental serotype K1/K2 and ST11 isolates and their isogenic mutants

**Fig. 3:**
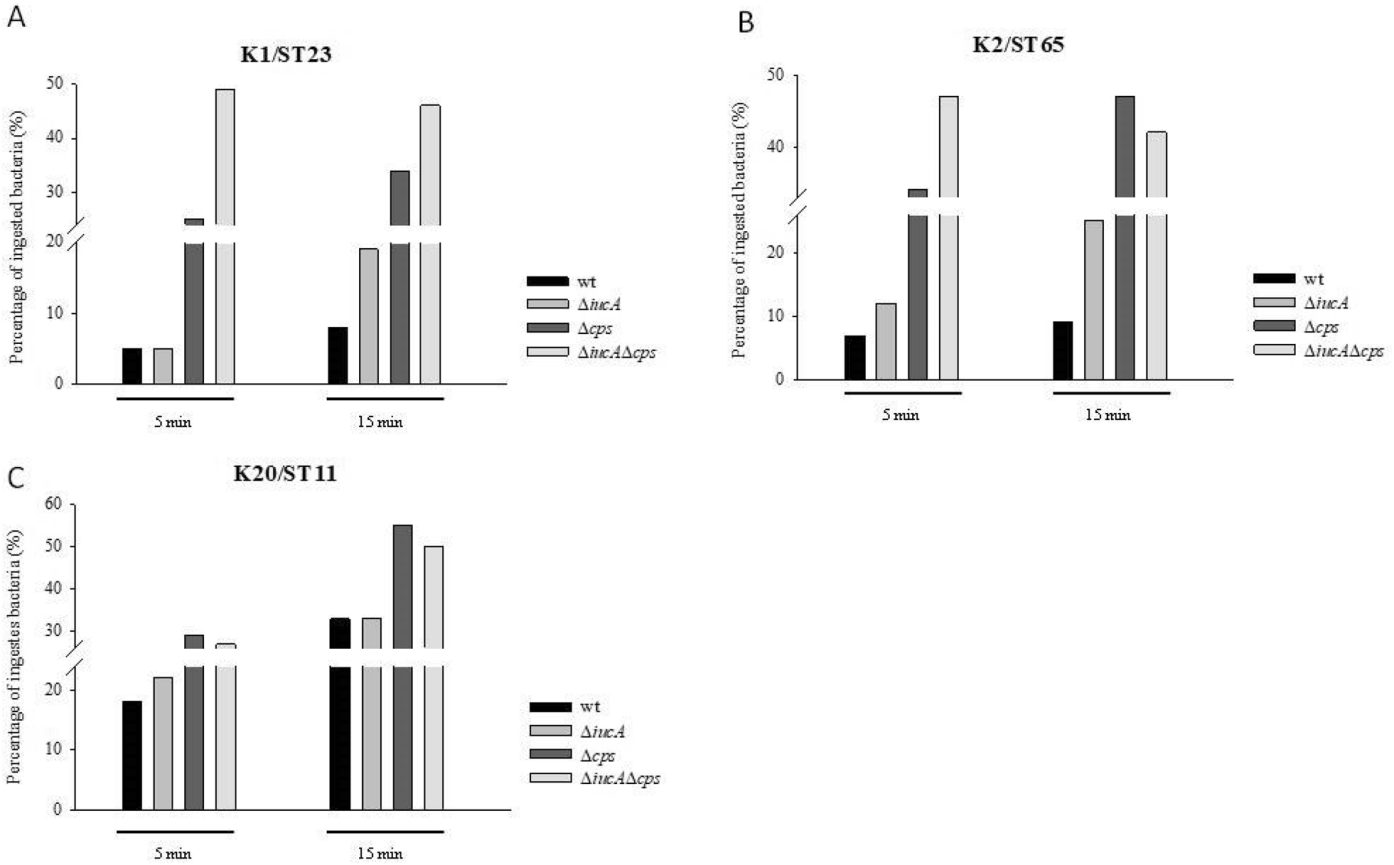
Neutrophil phagocytosis among HvKp-derived mutants from wild-type isolates. K1/K2 and ST11 isolates. The mutants included Δ*iucA,* Δ*cps* and Δ*iucA/*Δ*cps* double mutants

Compared to parental isolates, the *iucA*, *cps* and *iucA*/*cps* double mutants, all *cps* mutants were more susceptible to phagocytosis (Fig. 3A–C). Although aerobactin (*iuc*) was suggested to be an important factor contributing to virulence, Δ*iucA* mutants exhibited phagocytic resistance similar to that of their parental strains. The results obtained from the phagocytosis experiments indicated that *iuc* and *cps* played different roles in virulence. CPS played an important role in resistance to neutrophil phagocytosis, while *iuc* did not play a role in neutrophil phagocytosis.

For the serum resistance test, pooled normal human serum was collected from 10 healthy adults to examine the complement-killing effect on K1/K2 and ST11 isolates. Serotype K1/K2 strains and Δ*iucA* mutants showed more resistance (grade 5–6) to serum complement killing than other strains. CPS mutants and double mutants of Δ*iucA*/Δ*cps* did not show differences in susceptibility to serum complement killing. The Δ*cps* and Δ*iucA*/Δ*cps* mutants of serotype K1/ST23 and serotype K2/ST65, respectively, remained resistant to complement killing (grade 5–6). Clinical isolates of serotype K20/ST11 and their mutants were susceptible (grade 1) to serum complement killing. Since all 17 parental ST11 isolates were serum susceptible, the serum susceptibility of Δ*cps* and Δ*iucA* mutants of the remaining serotype K47 and K64 isolates were not determined (Table 3).

**Table 3:** Comparison of the phenotypic and virulence differences of one select isolate from each group of HvKp and their virulence gene knockout mutants

Serotypea	MLST	Strain	Serum assay: R, I, S (grade)	String test (≥ 5 mm)	LD_50_ (CFU)
K1	ST23	wt	R (6)	+	3.98 × 10^2^
		Δ*iucA*	R (6)	+	1.0 × 10^4^
		Δ*cps*	R (6)	− ve	≥ 10^7^
		Δ*iucA*Δ*cps*	R (6)	− ve	≥ 10^7^
K2	ST65	wt	R (6)	+	2.4 × 10^2^
		Δ*iucA*	R (5)	+	4.17 × 10^4^
		Δ*cps*	R (6)	− ve	≥ 10^7^
		Δ*iucA*Δ*cps*	R (6)	− ve	≥ 10^7^
K20^a^	ST11	wt	S (1)	− ve	≥ 10^7^
		Δ*iucA*	S (1)	− -ve	≥ 10^7^
		Δ*cps*	S (1)	− ve	≥ 10^7^
		Δ*iucA*Δ*cps*	S (1)	− ve	≥ 10^7^
K47^a^	ST11	wt	S (1)	− ve	≥ 10^7^
K64^a^	ST11	wt	S (1)	− ve	≥ 10^7^

^a^One isolate of each serotype K1, K2 and MLST11, was selected as a representative isolate for each group to generate virulent gene knockout mutants

**Fig. 4:**
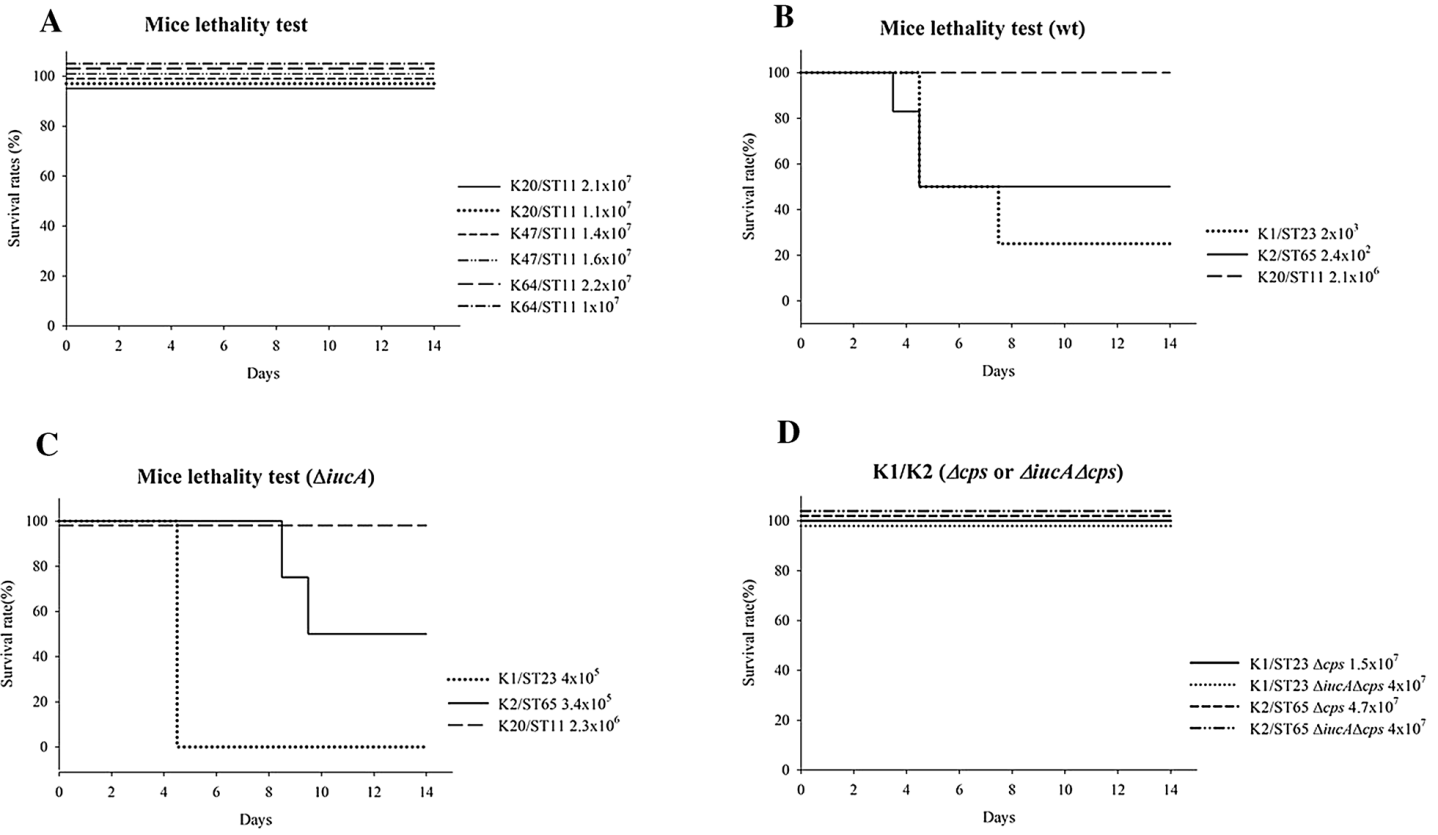
The mean lethal dose, i.e., the LD_50_, of wild-type HvKp isolates and their derived mutants in mice

Virulence was assessed in vivo in mice for 14 days via intraperitoneal (IP) injection of different inoculums of *K. pneumoniae* isolates and their derived mutants. Two of each ST11 isolate with serotypes K20, K47 and K64 were selected for comparison of virulence, and IP injection of all the selected ST11 isolates resulted in an avirulence phenotype (Fig. 4A). No difference in the virulence of ST11 isolates was observed for different serotypes. Thus, one isolate in this ST11 group was selected for further comparison to serotype K1/K2 isolates. Mice that received IP injections of K1/ST23 and K2/ST65 isolates showed significantly greater mortality than those that received K20/ST11 isolates beginning on day 5 post-injection (Fig. 4B). In the Δ*iucA* mutant study, Δ*iucA* had a lethal dose in mice that was decreased ~ 100-fold compared with that of the parental isolate (Fig. 4C). In the virulence gene knockout groups, including the *cps* and *iucA/cps* mutant groups, all mice survived injection of a high number of CFU (Fig. 4D), indicating that *cps* makes a major contribution to virulence. These results demonstrate that K1 and K2 were significantly more virulent than ST11 isolates and that relatively low concentrations of bacteria were sufficient to kill a significant number of the mice in the different groups. In addition, the virulence degree of ST11 isolates was comparable to that of *cps* mutants and Δ*iucA/*Δ*cps* mutants, and mice in both groups survived with no symptoms of infection. ST11 isolates might have similar degrees of virulence as virulence factor mutants, implying that the ST11 isolates in this study are avirulent.

In summary, according to assessments of virulence by different models (Table 3), ST11 was less virulent than the other strains in both the in vitro and in vivo models. The ST11 isolates were less susceptible to neutrophil phagocytosis and serum resistance and did not cause lethality in mice, even at high doses (10^7^ colony forming units). Although all parental strains, i.e., K1, K2 and ST11, including serotypes K20, K47 and K64, have been termed HvKp in previous publications, their virulence varied in different assessment models.

## Discussion

The term HvKp has been frequently used in recent publications. Many studies that used this term studied patients with severe infectious disease, such as the development of complications or mortality [1, 2, 4]. Through the collection of isolates from these patients, experimental work was performed on *K. pneumoniae* to determine the possible cause of the severe illness. In addition to patient factors, invasiveness and virulence are considered important bacterial factors affecting the severity of disease or clinical outcome [2, 4]. By comparing the isolates that cause severe illness with those that do not cause severe diseases, differences in the virulence of isolates can be identified by models used to assess virulence, and the factor(s) contributing factor/s to the difference in virulence can be determined; isolates that are identified as virulent through these methods were considered HvKp. In this study, we investigated isolates that were termed HvKp according to previously published descriptions and assessed these isolates with the same experimental platform. Serotypes K1/K2 and ST11 are the two groups that were frequently named HvKp in recent publications (Table 1). According to the virulence factors that were described previously, we selected isolates with similar virulence gene profiles in this study to minimize the difference in virulence degree between these two groups (Table 2). Serotypes K1 and K2 contained an extra *clbA* (colibactin) gene, and all other isolates, including 4 K2 isolates and 17 ST11 isolates, had identical profiles of the virulence genes *entB, iroN, iucA, iutA* and *rmpA,* but not *clbA*.

The string test has been widely used for initially screening virulent *K. pneumoniae*. Both serotypes K1/K2 were positive in the string test, while all ST11 isolates were negative. These results were consistent with previous studies showing that serotype K1/K2 isolates are generally more mucoid than other serotypes [4, 9, 19, 28]. The Δ*cps* mutant of serotype K1/K2 lost the mucoid phenotype, indicating that *cps* is involved in mucoviscosity. Since *cps* is highly correlated with neutrophil phagocytic resistance [26], it was expected that serotype K1/K2 isolates would have a higher resistance to phagocytosis than ST11. Previous studies have shown that serum resistance varies among *K. pneumoniae* isolates. Simoons-Smit et al. [29] observed that the loss of the K antigen (capsule) could enhance serum-mediated complement killing, while Tomás et al. found that the capsular polysaccharide seemed not to play any important role in resistance to serum bactericidal activity in this bacterium [30]. The serum resistance of different *cps* knockout strains was also reported in a previous study [30]. The inconsistencies among studies warrant further investigation.

In the mouse lethality test, the mean survival rate occurred when between 10^2^ and ≥ 10^7^ CFU was administered. Our previous criterion used to assess virulence was LD_50_, with ≤ 10^2^ CFU representing hypervirulence and ≥ 10^7^ indicating avirulence. The results in this study have shown that there is a large difference in the lethal dose in mice among HvKp isolates. K1/K2 isolates have an LD_50_ between ≤ 10^2^ and 10^3^ CFU, while mice show no symptoms of illness when challenged with serotype K20, K47 or K64/ST11 isolates at an inoculum of 10^7^ CFU, a saturation dose for IP injection. Since 4 serotype K2 isolates have an identical virulence gene profile to ST11 isolates, the extra *clbA* gene could not explain the large difference in virulence between serotype K1/K2 and ST11 isolates.

Aerobactin has been suggested to play an important role in virulence and is also a key biomarker of the enhancement of the growth and survival of HvKp [23, 31]. Our results demonstrated that the aerobactin mutant from serotype K1/K2 parental isolates decreased the LD_50_ in mice by 100-fold (Table 3 and Fig. 4). The virulence degree could drop from hypervirulence to virulence according to the criterion of virulence categorization [4]. Thus, aerobactin plays an important role in virulence and should be considered in patients infected with aerobactin-carrying *K. pneumoniae*. In this study, the contribution of virulence between capsular polysaccharides with specific serotypes and aerobactin was compared. In the mouse lethality test, *cps* was a major factor contributing to the degree of virulence. Loss of the capsule caused a substantial decline in the virulence of HvKp to avirulence (Fig. 4).

Previous studies used wax moth *Galleria mellonella* larvae to assess the virulence of the Kp pathogen. Since moth larvae have a short life span and easily reproduce in laboratory environments, their use as an in vivo model for quantitative studies is recommended [2, 32]. However, a comparative study of ST258 strains reported different degrees of virulence in mammals and non-vertebrates and indicated that a strain that resulted in rapid death in moth larvae was avirulent in the mice [33]. More studies have used mice to investigate human infectious diseases and to examine the lethality and toxicity response in rodents, and the results are reliable [23, 24, 34]. One clinical study from China investigated unknown Kp isolates collected from a local hospital. A mouse lethality test and serum assay revealed that only the K2 isolate was virulent (< 10^2^), and that the ST11 isolates were non-virulent (> 10^6^) [8]. The result was consistent with the findings of our present study.

To assess the virulence of bacteria, both in vitro and in vivo models can be adopted. Model selection depends on the factor being assessed and the reference used for the comparison. The choice of model becomes an important point to differentiate one strain from another. Different models have limitations in the magnitude of the factor that can be assessed or the degree to which the factor can be magnified to determine differences in virulence. Thus, the choice of an infection model may lead to over- or underestimation of virulence. In this regard, the term HvKp is used for two different types of *K. pneumoniae.* However, in determining causes of human infection, in vivo or animal models are considered better than in vitro models for assessing the level of virulence in humans.

Previous studies on HvKp used different in vivo models to assess extreme virulence, but those models were studied individually. Individual studies of HvKp were not problematic. Those works showed that HvKp had a higher level of virulence than the classic reference *K. pneumoniae*. After the two different types of HvKp were injected into the same animal model, the level of virulence was different. Although HvKp has been described frequently in different studies, the virulence was notably different when assessed in the same animal model. Since animal models more closely reflect the human condition than other models, we suggest that the term hypervirulence be based on the results of experiments in animal models instead of other models to avoid confusion of the virulence of *K. pneumoniae*. Virulence and non-virulence could be used in a relative manner, especially in comparison studies.

## Conclusion

Although serotype K1/K2 and MLST-11 K*. pneumoniae* have both been frequently described as "hypervirulent *K. pneumoniae*" in the previous literatures, the virulence level of MLST-11 K*. pneumoniae* was substantially lower when assessed in the same animal model. The term "hypervirulent *K. pneumoniae*" should be carefully used to avoid confusion.

## Supplementary Information

**Additional file 1: Tabls S1.** PCR primer sets for virulence gene detection and serotyping.** Table S2.** Primers used for in-frame deletion of iucA and wza.

## Data Availability

The raw data involved in the study will be provided upon request. Bacterial strains will be provided for academic use only, and transfer must be approved by the safety department.
