# Supplementary material for: Virulence among different types of hypervirulent Klebsiella pneumoniae with multi-locus sequence type (MLST)-11, Serotype K1 or K2 strains
Source: Gut Pathog. 2021 Jun 22;13:40. doi: 10.1186/s13099-021-00439-z (PMC8218402; doi:10.1186/s13099-021-00439-z)
Supplement: Supplementary file 1 — Additional file 1: Tabls S1. PCR primer sets for virulence gene detection and serotyping. Table S2. Primers used for in-frame deletion of iucA and wza. [file 13099_2021_439_MOESM1_ESM.docx]

Supplementary Table 1. PCR primer sets for virulence gene detection and serotyping

| Gene | Primer name | Primer sequence (5’-3’) | Product size (bp) |
| --- | --- | --- | --- |
| clbA | ClbA 1F | ATGAGGATTGATATATTAATTGGACA | 735 |
|  | ClbA 1R | TCAATTCTGCCCATTTGACG |  |
| entB | entB-F | ATTTCCTCAACTTCTGGGGC | 371 |
|  | entB-R | AGCATCGGTGGCGGTGGTCA |  |
| iroN | iroN-F | GTCCGGCGGTAACTTCAGCC | 829 |
|  | iroN-R | TCAGAATGAAACTACCGCCC |  |
| iucA | iucA-F | ATAAGGCAGGCAATCCAG | 2927 |
|  | iucA-R | TAACGGCGATAAACCTCG |  |
| iutA | iutA-F | GGCTGGACATCATGGGAACTGG | 300 |
|  | iutA-R | CGTCGGGAACGGGTAGAATCG |  |
| rmpA | rmpA-F | TACATATGAAGGAGTAGTTAAT | 505 |
|  | rmpA-R | GAGCCATCTTTCATCAAC |  |
| wzyK20 | wzyK20F | CGG TGC TAC AGT GCA TCA TT | 741 |
|  | wzyK20R | GTT ATA CGA TGC TCA GTC GC |  |

Supplementary Table 2. Primers used for in-frame deletion of *iucA* and *wza*.

| Primer name | Primer sequence (5’-3’) |
| --- | --- |
| Xbal-AF | AGT CTA GAG CTG TTC AGC GAA CAT TAT CGC |
| iucA-AR | CAA AAA TTA TTA GGA TTG ATA AAA TTT ATT TTA TAA ATA AGC |
| iucA-BF | GCT TAT TTA TAA AAT AAA TTT TAT CAA TCC TAA TAA TTT TTG ATG TCT AAG GCA AAC ATC GTT CAC |
| Sacl-BR | TCG AGC TCG TGA TTC ATT ACA GAC CGA CCT CC |
| iucA-IN-F | GAT GTG GCT GCA CAG TGC TTC |
| iucA-IN-R | TAG CGT TCG GCC TCC TGC TG |
| K2wza-AF1 | ACT CTA GAG GCA GTA CCA AAT CTC CGC TAG |
| K2wza-AR1 | AAT GTC ACA TCA TCA GTA AAT CAA AAT TTG |
| K2wza-BF1 | CAA ATT TTG ATT TAC TGA TGA TGT GAC ATT TAT GTT TAG TAC AAT ATT AAT TGT TTG CAC AG |
| K2wza-BR1 | ACG AGC TCT TAA CCT TGC CCA TCC ACG C |
| K2wza-IN-F | CAA TGT GTA CCC GAT GAC GC |
| K2wza-IN-R | CCA GTC AGC ATC TGG TGC AA |
| wza-A-F | CGG GGT ACC GCG GAG ACT CAA TTT GCG TCA GC |
| wza-A-R | TTG CAG ATC ACC AAC TGG CG |
| wza-B-F | CGC CAG TTG GTG ATC TGC AAG CCG ATT ACC AAC GCT AAT GCC G |
| wza-B-R | GCT CTA GAA TGC CAT GCG GCC AAT GAC TG |
| wza-S-F | CCT GTC GCA TCA CTC AAT GC |
| wza-S-R | TGT TAG CAC GAC CAA ACC CG |

Restriction enzymes *KpnI* and *XbaI*
